# Improving the Quality of Oocytes with the Help of Nucleolotransfer Therapy

**DOI:** 10.3390/ph14040328

**Published:** 2021-04-02

**Authors:** Michal Benc, Frantisek Strejcek, Martin Morovic, Alexandra Bartkova, Matej Murin, Ahmed Gad, Amelie Bonnet-Garnier, Florina Popovska Percinic, Jozef Laurincik

**Affiliations:** 1 Faculty of Natural Sciences, Constantine the Philosopher University in Nitra, Nabrezie mladeze 91, 94974 Nitra, Slovakia; benc.michal@gmail.com (M.B.); mmorovic@ukf.sk (M.M.); alexandra.bartkova@ukf.sk (A.B.); laurincik@gmail.com (J.L.); 2 Institute of Animal Physiology and Genetics, The Czech Academy of Sciences, 27721 Libechov, Czech Republic; murin@iapg.cas.cz (M.M.); gad@iapg.cas.cz (A.G.); 3 Université Paris-Saclay, UVSQ, INRAE, BREED, 78350 Jouy-en-Josas, France; amelie.bonnet-garnier@inrae.fr; 4 Ecole Nationale Vétérinaire d’Alfort, BREED, 94700 Maisons-Alfort, France; 5 Faculty of Veterinary Medicine, St. Cyril and Methodius University in Skopje, 1000 Skopje, North Macedonia; florinap@fvm.ukim.edu.mk

**Keywords:** oocyte, embryo, zygote, nucleolus, nucleolotransfer

## Abstract

The nucleolus is an important nucleus sub-organelle found in almost all eukaryotic cells. On the one hand, it is known as a differentiated active site of ribosome biogenesis in somatic cells, but on the other hand, in fully grown oocytes, zygotes, and early embryos (up to the major embryonic genome activation), it is in the form of a particular homogenous and compact structure called a fibrillar sphere. Nowadays, thanks to recent studies, we know many important functions of this, no doubt, interesting membraneless nucleus sub-organelle involved in oocyte maturation, embryonic genome activation, rRNA synthesis, etc. However, many questions are still unexplained and remain a mystery. Our aim is to create a comprehensive overview of the recent knowledge on the fibrillar sphere and envision how this knowledge could be utilized in further research in the field of biotechnology and nucleolotransfer therapy.

## 1. Introduction

Nucleoli in somatic and stem cells, and also in growing oocytes and early embryos after the major genome activation, have a typical structure composed of fibrillar centres, dense fibrillar components, and granular components [1,2]. These differentiated nucleoli are involved in many cell processes, i.e., ribosomal RNA synthesis [3], chromosome segregation, cell cycle regulation, and many others [4,5]. However, the situation differs in fully grown oocytes and in the early stages of embryonic development up to the major genome activation [6]. Nucleoli at the end of the oocyte growing phase lose their typical differentiated structure and are composed of compact homogenous fibrillar material [7,8]. Based on their compact fibrillar structure, these nucleolar structures are also known as “fibrillar spheres”. As the oocyte nucleoli become uniformly fibrillar, RNA synthesis decreases and the chromatin is separated from the nucleoli [9,10]. The fibrillar sphere is first visible in the germinal vesicle (GV) stage, and disappears during the progress of oocyte maturation. Later on, i.e., after fertilization, it is visible again in the zygote and early embryos until major genome activation. Fibrillar nucleoli present in the GV stage of fully grown oocytes are called a nucleolus-like body (NLB) [11]. The same structure as an NLB, which usually appears in zygote’s pronuclei (PNs) shortly after the fertilization, is called a nucleolus precursor body (NPB) [12]. It can be said that NLBs and NPBs are very similar in many ways. This opinion is supported by studies with NLB transfer, when a previously enucleolated oocyte was unable to re-build NPBs in PNs [13]. This means that NPBs in PNs are rebuilt from the same components as NLBs in a germinal vesicle. Some small differences are based on the developmental stages in which the specific fibrillar sphere (NLB or NPB) is present. As is mentioned below, an NLB has a closer association with chromatin than was observed in NPBs. On the other hand, NPBs form the building blocks for differentiated and fully functional nucleoli [14]. It is appropriate to note that many functions are still unknown, and could be studied with the help of proteomic and transcriptomic profiling or with functional nucleolotransfer studies.

## 2. Fibrillar Sphere Handling

Manipulations with NLB/NPB can be performed using micromanipulation techniques, with GV stage oocytes and zygotes being the most suitable developmental stages for this. However, it is only possible to manipulate with an NLB/NPB during the specific phases in which the NLB/NPB is visible. As was mentioned above, the NLB is present in fully grown oocytes isolated from antral follicles. The duration of the GV stage is species-specific, and can be extended by inhibiting oocyte meiotic resumption [15,16,17]. If oocyte maturation continues, the germinal vesicle is broken down in a process known as germinal vesicle breakdown (GVBD), and the NLB is disassembled during this process. However, the fibrillar sphere appears again as an NPB in PNs after fertilization or parthenogenetic activation. NPBs are reconstructed from components which were disassembled before. Their presence in the oocyte during the maturation process is ensured by intrinsically asymmetrical oocyte meiotic division [18].

The process of NLB/NPB isolation—enucleolation, was described for the first time in 2003 by Fulka’s team [19]. Enucleolation by micromanipulation is a unique technique, in which the NLB/NPB is removed without damaging the oocyte/zygote. In addition, the NLB/NPB is enveloped with cytoplasm during this process. This cytoplasmic cover protects the NLB/NPB from its dissolution in the culture medium. A detailed description of the enucleolation process was summarized in several studies [20,21].

The process of enucleolation does not cause irreparable damage to the chromosomes for further development [14,22]. It is probably caused by chromatin separation from the nucleoli during the changes at the end of the oocyte growing phase. Hand in hand, rDNA is extruded from the NLB during the transcription silencing [9,10]. However, according to our recent experience and 3D fluorescence in situ hybridization (3D FISH) analyses, we admit that short-term changes in chromatin distribution are possible (Bonnet-Garnier and Benc, in preparation).

In this regard, if the growing oocyte is enucleolated, actinomycin D has to be added into the culture medium in order to inhibit polymerase I and polymerase II activity, and also to support chromatin separation from the NLB. Additionally, in oocytes treated with actinomycin D, the NLB is better compacted for further enucleolation [23].

It has been found that the NLB is not essential during the maturation process from the GV stage to the metaphase II (MII) stage. A previously enucleolated fully grown oocyte matures into the MII stage in in vitro culture and can be fertilized or parthenogenetically activated. Moreover, the enucleolated oocyte is able to form pronuclei; however, without NPBs [13]. It was later found that if zygotes have pronuclei without NPBs as a result of oocyte enucleolation, it leads to centric and pericentric chromatin collapse [24,25]. However, if the NLB is re-injected into a previously enucleolated oocyte during any stage of maturation before fertilization, successful embryonic development is renewed [13]. On the other hand, if NPBs are enucleolated only a few hours after fertilization, there is no negative impact on embryonic development. This indicates that NLB is not necessary for the process of oocyte maturation; however, it is definitely essential immediately after fertilization [26].

NPBs can be removed from PNs in a similar way to the NLB from a germinal vesicle. This enucleolation is more difficult, because micromanipulation with two pronuclei is needed. Sometimes the NPB is fragmented into several smaller NPBs, which complicates the enucleolation process even more. If the NPB is only removed from one pronucleus, embryonic development continues; however, the observed blastocysts are smaller and without prominent inner cell mass (ICM). It does not matter which PNs (male or female) are enucleolated [27].

The re-injection process of a previously enucleolated NLB/NPB is very similar to intracytoplasmic sperm injection (ICSI). In our experience, and as was mentioned in Kimura’s study on ICSI, a slight cooling of micromanipulation drops is helpful for successful NLB re-injection [28].

Incidentally, it is sufficient to inject the NLB into the cytoplasm of the oocyte. Additionally, it is possible to introduce more than one NLB. When that is done, oocyte maturation and embryonic development is successful. The opposite situation, when a smaller amount of NLB is re-injected or part of the original NLB is removed, leads to unsuccessful embryonic development [29]. If multiple NLBs/NPBs are injected, or handling with more than one NLB/NPB is needed, their ability to fuse into a single giant nucleolus can be utilised [30]. Handling one giant nucleolus is much easier than several NLBs/NPBs.

After the re-injection, the NLB/NPB is disassembled in the cytoplasm and transferred into the GV or PNs. This process is not well understood, however it is probably associated with nuclear proteins known as nuclear localization signals. These proteins provide nucleolar protein transfer through the nuclear membrane [31], and this transfer is clearly visible during the interspecies nucleolotransfer of NLB from the mouse oocyte to bovine oocyte. It is well known that the bovine oocyte has NLBs in their GV, which are, however, too small for visualisation under a light microscope [32]. If the mouse NLB is injected into the bovine oocyte, it is subjected to the same mechanism as the one that occurs during intraspecies nucleolotransfer. Over time, it is possible to observe the large mouse NLB in the bovine germinal vesicle instead of the typical “empty” bovine GV (Fulka and Benc, in preparation).

**Figure 1:**
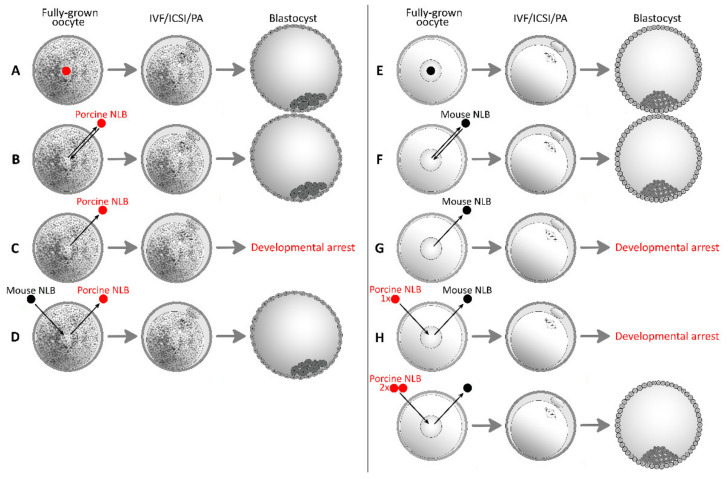
Intraspecies and interspecies nucleolus-like body (NLB) transfer. (IVF/ICSI/PA) In vitro fertilization, intracytoplasmic sperm injection, parthenogenetic activation; (**A**–**D**) porcine oocytes, blastocyst; (**E**–**H**) mouse oocytes, blastocyst; (**A**,**E**) intact group; (**B**,**F**) enucleolated and intraspecies re-injected oocytes develop to the blastocyst stage [13]; (**C**,**G**) embryos from previously enucleolated oocytes arrest before genome activation, porcine embryos usually at the 4-cell stage (**C**), mouse embryos usually at the 2-cell stage (**G**) [13]; (**D**,**H**) an NLB from a different species is able to support the embryonic development of an embryo from a previously enucleolated oocyte, but the amount of NLB material is essential. Significant differences in protein content were observed [30,35]. Two porcine NLBs are able to replace one mouse NLB. If one mouse NLB is replaced with one porcine NLB, embryonic development fails [33,34].

For a long time, attention was focused on intraspecies nucleolotransfer, and the first studies with interspecies nucleolotransfer among distant species were published only recently, opening up great opportunities for further research. It was observed that NLBs are not qualitatively different between species when a porcine NLB was replaced with a mouse NLB and vice versa (Figure 1D,H) [33,34]. On the other hand, quantitative differences in relative protein concentrations (RPC) in mouse and porcine NLBs were observed, in which mouse NLBs had approximately twice the total amount of protein of porcine NLBs [30,35]. However, this deficit can be compensated for, as is described in Figure 1H [34]. This behaviour corresponds with findings from intraspecies nucleolotransfer, when part of the NLB was removed from the oocyte and the subsequent embryonic development became considerably endangered. However, if more than two extra NLBs were added into the oocyte, worse results in terms of embryonic development were observed, but the reason for this has not been explained yet [29]. There is probably an optimal amount of nucleolar proteins important for embryonic development that is species-specific. On the other side, limit values exist, and overcoming them can lead to abnormal embryonic development.

A recent breakthrough study significantly supports these findings. When authors tried to recover NLB functionality by exogenous Npm-2 mRNA injection into previously enucleolated oocytes. Surprisingly, their experiment was successful, and embryonic development was renewed [25]. Npm-2 is a dominant nucleolar protein present in the nucleus. Its absence leads to the disruption of NLB. Therefore, if it is missing in oocytes, NPBs are not formed in early embryos. This causes several complications such as chromosome segregation defects and the disorganization of chromatin [36]. Basically, the absence of Npm-2 corresponds to oocyte enucleolation, and its absence is responsible for several irreversible changes at the chromatin level. This is in line with an optimal amount of nucleolar proteins theory.

For better visualisation of the data from nucleolotransfer, we summarize the embryonic developmental success of embryos derived from enucleolated and re-injected oocytes (Table 1).

## 3. NLBs/NPBs and Their Impact on Oocyte Maturation and Embryonic Development

The first study on the relationships between the fibrillar sphere and embryonic developmental success was Tesarik’s work in 1999 [37], in which the authors observed the number of NPBs, their distribution, and size in a human zygote’s pronuclei after in vitro fertilization (IVF). The relationship between these parameters in the maternal and paternal pronucleus and further embryonic development was confirmed. On the one hand, the same distribution, number, and size of NPBs predicted successful embryonic development and chances for pregnancy, on the other hand, the embryonic development of zygotes with abnormalities in these parameters was significantly reduced. Later, these results were supported by Gianaroli [38]. Recently, these results were confirmed by a study of human early embryos (159) obtained from an assisted reproduction clinic, where zygotes with a central position and frontal alignment of PNs with 7–8 NPBs were considered to be the embryos with the highest quality, with an increased ability to reach the blastocyst stage (Morovic and Jedlickova, in preparation).

**Figure 2:**
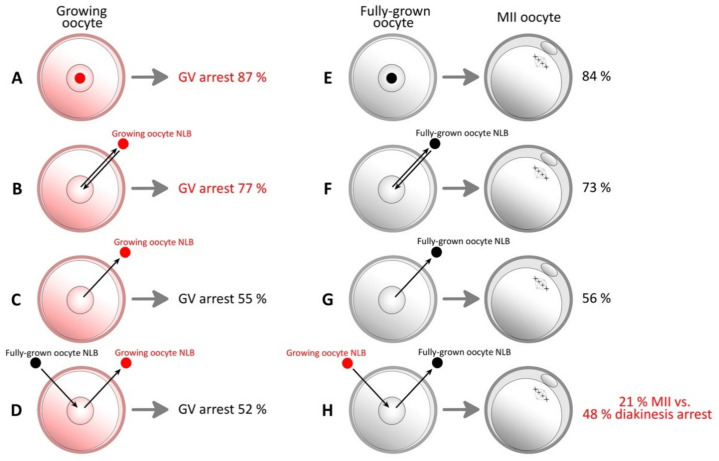
Enucleolation and re-injection of growing and fully grown oocytes. (**A**,**E**) Intact group. A growing oocyte has limited meiotic competence during the in vitro maturation, and its maturation fails. On the other hand, a fully grown oocyte matures to the metaphase II (MII) stage without any problems; (**B**,**F**) enucleolation and re-injection back into oocytes does not have a significant effect on maturation rate compared to the control group; (**C**,**G**) NLB removal from a growing oocyte has a significant impact on maturation rate. Along with that, the inhibitory effect is also eliminated. Results in fully grown oocytes correspond with previous observations [13]; (**D**,**H**) when the growing oocyte NLB is replaced with an NLB from a fully grown oocyte, the results were very similar to enucleolated growing oocytes. Surprisingly, when a fully grown NLB is replaced with an NLB from a growing oocyte, the maturation rate drops, and 48% of oocytes were arrested in diakinesis. This supports the idea that the NLB from growing oocytes has an inhibitory effect on oocyte maturation [41].

As was mentioned above, during the early stages of oogenesis, nucleoli in oocytes resemble fully differentiated nucleoli similar to somatic cells, with typical structure and functions [1,2]. When oocytes become arrested in the first meiotic prophase of the diplotene stage, they enter the growing phase. During the oocyte growing phase, nucleoli are also subject to structural and functional changes and begin to resemble the NLB [39]. At the end of the growing phase, oocytes reach the fully grown stage [40]. These fully grown oocytes are able to reach metaphase II. It is important to note that growing oocytes have limited meiotic competence during the in vitro maturation, and they are unable to overcome GVBD. However, if the growing oocyte NLB is removed from the oocyte, it is able to overcome GVBD [41]. This is a very important observation, especially in the context of the knowledge that NLB is not necessary for maturation [13]. That means that the NLB from growing oocytes has an inhibitory effect on oocyte maturation, and indicates a relationship between the growing oocyte NLB and meiotic resumption [19]. This conclusion was confirmed by a study in which the growing oocyte NLB was re-injected back into the previously enucleolated growing oocyte, and for this reason, meiotic resumption was renewed, and the oocyte remained arrested at the GV stage [41]. Additionally, an NLB from a growing oocyte causes meiotic resumption of a fully grown oocyte. This effect is increased if the original NLB is removed from a fully grown oocyte. The authors consider that a growing oocyte NLB is involved in the meiotic arrest and has an inhibitory effect on oocyte maturation. On the other hand, according to this study, NLBs of fully grown oocytes probably lose this ability (Figure 2). This means that the initial state of the NLB is crucial for oocyte maturation.

There are several ways to identify a growing oocyte. Morphological evaluation can be included among the basic methods. However, there are some more precise ways to select growing and fully grown oocytes. One of the best techniques is vital staining with brilliant cresyl blue dye (BCB). This staining method is based on glucose-6-phosphate dehydrogenase (G6PDH) activity. The BCB dye is degraded by the enzyme’s activity [42]. The G6PDH enzyme is exclusively synthesized in growing oocytes. Therefore, growing oocytes with high G6PDH activity are unstained (BCB^−^), while the fully grown oocytes are deep blue (BCB^+^) [43]. Thanks to this, it is possible to select growing and fully grown oocytes before their in vitro maturation (IVM). In our pilot experiments, we compared the relative protein concentration in NLBs of BCB^+^ and BCB^−^ oocytes, and, surprisingly, no significant differences were found in BCB^+^ and BCB^−^ oocytes in terms of RPC [35]. These data suggest that the quantity of proteins in oocyte nucleoli has already been established in growing oocytes. We expect more data after evaluating the proteomic profile with the help of mass spectrometry [44]. However, it has to be noted that a relatively large number of high-quality NLBs are needed [18,25].

Besides the above differences, there is another important factor in embryonic development, which is the nucleolus–chromatin association. There are two forms of nucleolus–chromatin association in oocytes in the GV stage. We distinguish them according to their chromatin condensation around the NLB. The surrounded nucleolus (SN) form is characterised by an intimate association between the NLB and chromatin, while the non-surrounded nucleolus (NSN) form is characterised by no close association between chromatin and the NLB [45,46]. Chromatin condensation in the GV stage has a very significant impact on later embryonic development [47]. It is well known that chromatin condensation is closely connected to the growing phase of the oocyte. Gradually, RNA synthesis decreases, and the chromatin separates from the nucleoli. Therefore, NSN oocytes are mostly growing and, vice versa, SN are mostly fully grown. At the end of the growing phase, the NSN configuration is changed to SN in a process known as the non-surrounded nucleolus to surrounded nucleolus transition. Although we still do not know the exact role of the NLB in this process. Currently known results suggest that these interactions are not accidental, and are probably related to the “maturity” of the NLB [41]. On the other hand, there is a different chromatin association around the NPBs in zygotes. Interactions similar to NLBs have not been detected, therefore the functions of NLBs and NPBs change over time. This was confirmed by a study in which embryonic development was supported by nucleoplasmin 2 (Npm-2) instead of NPBs. This confirms that there must be an alternative mechanism in early embryos, which are able to substitute NPB [27,36]. Therefore, we believe that improving the quality of the oocytes by intra- or interspecies nucleolotransfer, or by adding key components into the oocytes, can be potentially used in assisted reproduction or in recovering endangered species.

As we can see, the flow of technical and findings development in this field is extensive. For this reason we summarize the gradual development and milestones in microsurgical approaches (Table 2).

## 4. Preservation of NLB/NPB

Preservation plays a key role in assisted reproductive technologies (ART). In practice, cryopreservation is used for the long-term storage of sperm, oocytes, or embryos [48,49,50]. It is a valuable way to save time and increase the chances for pregnancy. Embryos prepared by IVF techniques that are not used immediately can be stored for further use by cryopreservation. Due to this, cryopreservation also has an ethical dimension.

Many fields of embryotechnology have been improved thanks to this technology. Therefore, the new question arises: is it possible to store the NLB/NPB, and should this be used in practice?

There are several ways in which an NLB/NPB can be stored. We mentioned above that during the enucleolation process, the NLB/NPB is removed from the oocyte/zygote and also enveloped with cytoplasm. This complex is usually called a “nucleoloplast”, and the cytoplasmic cover protects the NLB/NPB from dissolution in culture medium [20,21]. A nucleoloplast prepared in this way lasts for several hours, depending on the osmotic pressure, temperature, or type of the medium. However, in this case the cytoplasm and the origin of the species play crucial roles. For example, in our experience, a mouse nucleoloplast is more resistant than a porcine one. When enucleolated NLBs enclosed in nucleoloplasts were stored for 26 h, approximately half of them were lost over that period. For better localisation, they were finally inserted into the empty zona pellucida; however, not even this prevented nucleoloplast disintegration, suggesting that this technique is not suitable for the long-term preservation of NLBs/NPBs.

As another option, it is possible to store the NLB/NPB for several days without damage in a high-percentage polyvinylpyrrolidone (PVP) solution [20,51]. However, it is important to note, that the cytoplasm from the nucleolus has to be removed from the NLB/NPB before its transfer into the PVP. NLB/NPB creates a thin shell in PVP, which subsequently protects them. Whilst there are several types of PVP, it is important to choose one with a high molecular mass.

However, the preservation of NLB/NPB for several days is still not enough for assisted reproduction or biotechnology. For this purpose, it is appropriate to mention work on the cryopreservation of nuclear material instead of the whole oocyte. The cryopreservation of oocytes is a well-known method, which is routinely used in human or animal assisted reproduction. However, some species tolerate this method better than others. In particular bovine or porcine oocytes are highly sensitive, and their storage is still unsatisfactory. The main reason for this seems to be a high proportion of lipid structures in the cytoplasm [52,53]. However, a clever solution to this problem was the cryopreservation of nuclear material instead of whole oocytes. In some studies, the germinal vesicles were removed from the oocytes by micromanipulation techniques, and these karyoplasts were then cryopreserved by vitrification. After thawing, successful transfer into the cytoplast was performed. Additionally, reconstructed oocytes were able to mature to the MII stage [54,55,56]. A valuable insight is that GVBD does not occur in karyoplasts. This is probably caused by the limited amount of cytoplasm and key cell cycle factors [57]. Therefore, it seems that this approach could be used for the cryopreservation of NLBs/NPBs. Based on knowledge from our interspecies nucleolotransfer experiments, donor oocytes from another species can be used for NLB/NPB reconstruction after thawing. This information could be valuable for improving the quality of oocytes during work with endangered species.

## 5. NLB/NPB Transfer and Its Perspective in Therapy

Many methods in research or in clinical practice are stopped because of legal or ethical standards. One of the most important aspects of NLB/NPB handling is not coming into conflict with these aspects. Handling NLBs is not an issue, because they can be obtained directly from the female gamete. The situation with NPBs, which are present in a zygote’s PNs, is a little more complicated. On the one hand, the enucleolation process does not destroy the zygote, but on the other hand, an embryo without NPBs is doomed. This could be a problem for work with human embryos. Fortunately, it is well known that the NLB/NPB is strictly maternally inherited and does not contain the DNA. As was mentioned above, NLBs and NPBs are very similar, and in many ways the same. Our assumption is substantiated by several findings. First, the NPBs in zygotes are reconstructed from the same material as the NLBs in fully grown oocytes because, if the NLB is removed from oocyte, the NPBs are not formed in early embryo [13]. Second, NLBs and also NPBs are composed only of fibrillar material. There are no other component differences between them [58,59]. Therefore, it is not necessary to prepare a fully featured embryo by fertilization and, potentially, embryos with NPBs are not required. It is enough to improve the oocytes quality by NLB transfer before fertilization, without the prior creation of a new life [60].

Another positive thing is that nucleolotransfer is not dependent on the oocyte maturation stage. Some diseases in human assisted reproduction, for example, mutated mitochondrial DNA (mtDNA), can be repaired by the micromanipulation transfer of a karyoplast into a donor oocyte with undamaged mtDNA [61]. During this and similar transfers, an identical oocyte maturation stage is required, but the NLB can be transferred at any time [57,62,63]. It is crucial to note that this statement applies to the maturation stage of the oocyte whose quality we would like to improve by nucleolotransfer. As mentioned above (Figure 2), the origin of the NLB is extremely important in this case, because an NLB from a growing oocyte has an inhibitory effect on oocyte maturation [41]. This would, of course, be counterproductive. On the other hand, as we mentioned, the oocyte maturation stage is not important. This is advantageous for nucleolotransfer because it opens up a wide range of possibilities for its use, and allows for the choice of oocyte maturation stage, which depends purely on the type of problem that needs to be solved.

In human assisted reproduction and biotechnology, fully grown oocytes are preferred for their developmental competence. Growing oocytes and low-quality fully grown oocytes are usually discarded. Previous studies have tried to find the best way to use these inappropriate oocytes [43]. If possible, many infertile couples could find their way to having their coveted child, many endangered species could be saved, and many methods in biotechnology could be improved. We would like to draw attention to nucleolotransfer as one possible way to achieve these things. In our opinion, oocyte maturation and embryonic development could be improved by nucleolotransfer. A pilot study with patients whose pregnancy repeatedly failed showed that adding key components can increase their chance to have a baby. When authors transferred the cytoplasm from a donor’s oocytes to the recipient’s oocytes, the quality of those oocytes was increased. Its improvement could be caused by an increase in important and necessary factors and organelles such as mitochondria, etc. [61]. It is likely that adding an extra NLB/NPB, especially from a fully grown oocyte, could have a similar impact on oocyte maturation and embryonic development. It was confirmed that adding 1–2 NLBs/NPBs does not have a negative impact on embryonic development. On the contrary, the compaction of a mouse embryo with an extra NLB is faster, and it is launched sooner. Tighter junctions between the blastomeres were already observed during the 4-cell stage. It is important to note that there is probably an optimal amount of nucleolar proteins important for embryonic development, which is species-specific (Figure 2).

For a long time, the NLB/NPB was considered to be only a material repository. This perception was caused by NLB inactivity during fully grown oocyte maturation. Additionally, the removal of NLB does not disrupt chromosome segregation or spindle assembly [13]. On the other hand, many studies support evidence that NPB’s presence in zygotes is essential [13,22]. Not only because of its material repository function, but also for NPB’s crucial role in processes at the chromatin level [64]. Its absence leads to abnormal centromere distribution. Under normal circumstances, the centromeres are localized in close proximity to the NPB [65]. As was shown in the absence of NPB, these regions are scattered in the nucleoplasm, which leads to embryonic development failure [66]. Additionally, it is important to note that rRNA transcription was already detected in the zygote [67]. This means that NPB becomes reactivated immediately after the fertilization. When Ogushi and Saitou injected NLB into the previously enucleolated oocytes before and after the fertilization, the embryos that originated from oocytes re-injected before fertilization reached successful embryonic development, whereas the embryonic development of oocytes re-injected after the fertilization was disturbed [24]. Kyogoku’s findings also confirm this assumption [26]. When they removed NPBs from early and late zygotes, enucleolated early zygotes did not reach successful embryonic development, while the late enucleolated zygotes reached the blastocyst stage. This suggests that NPBs start to function very soon after fertilization.

In the light of recent studies, this previous theory about material repository has been refuted and new roles of the NLB/NPB were observed. This leads to the assumption that the NPB plays a much more important role in early embryonic development as it has been accepted so far.

Additionally, our recent research showed that the NPB may also play a role in genome activation. The qPCR analysis of interspecies nucleolotransfer embryos showed that the NPB probably causes a time shift, depending on species origin. Major genome activation in mouse embryos initiates during the late 2-cell stage and in porcine embryos during the late 4-cell stage. When a mouse NLB was replaced with a porcine one, genome activation was shifted to the late 4-cell stage, as is typical in porcine embryos [34]. Possible links between NPBs and genome activation are in accordance with findings that NPBs and pericentromeric heterochromatin undergo necessary reorganization before embryonic genome activation [68,69].

All these data suggest that the NLB/NPB and their changes can have a huge impact and significance on the success of embryonic development, and could be applied in further therapy. It is important to note that if microsurgical methods such as nucleolotransfer become a validated ART technique or technique used in biotechnologies, it would be used in very specific cases. However, on the other hand, any improvement in this field is very important and welcome. In summary, we believe that nucleolotransfer can improve both oocyte maturation rates and early embryonic development. Additionally, since the NLB/NPB is not qualitatively species-specific [34], there is also the opportunity to take advantage of interspecies nucleolotransfer (xenotransplantation) in recovering endangered species or in ART, when the exact composition of NLB/NPB will be known and all current and future legal and ethical issues will be solved.

## 6. Conclusions

The role of the NLB/NPB in oocytes and embryos was, to a large extent, unknown for a long period of time. Since the discovery of enucleolation, a new era has begun in terms of understanding the various new functions of this structure, which strengthen its role in early embryonic development. In this article, we present knowledge on NLBs and NPBs and their possibilities and promise for improving the quality of oocytes in biomedical research and subcellular therapy. Micromanipulation techniques are an integral part of human assisted reproduction, and we have been shown many times that various genetically determined problems can be solved using them. Along with improving knowledge on NLBs/NPBs, we believe that not only nuclear transfer, but also sub-organelle nucleolotransfer will become one of the many techniques that are utilised to improve early embryonic development. As explained above, there are many ways in which NLBs/NPBs might be utilized in the near future.

## Figures and Tables

**Table 1:** In vitro development of embryos derived from enucleolated and re-injected oocytes.

Combination		Total Number of Oocytes	Total Number and % of Oocytes Forming Blastocyst, [Citation]
Oocyte	NLB		
Mouse	Mouse	123	62 (50%) [34]
Mouse	Porcine (2 NLBs)	281	126 (45%) [34]
Porcine	Porcine	194	72 (37%) [13]
		313	37 (12%) [33]
Porcine	Mouse	327	44 (14%) [33]

**Table 2:** Fibrillar sphere handling and finding milestone.

Year of Publication	Authors	Key Findings	Species
2003	Fulka J. Jr. et al.	First successful microsurgical enucleolation of NLB from the oocyte [19].	Pig
2008	Ogushi et al.	The maternal NLB is essential for early embryonic development in mammals [13].	Mouse, pig
2010	Ogushi and Saitou	The nucleolus in the oocyte is required for the early step of both female and male pronucleus organization [24].	Mouse
2011	Kyogoku et al.	NLB from growing oocytes has an inhibitory effect on oocyte maturation [41].	Pig
2012	Fulka H. et al.	First production of giant nucleolus and relative protein content analyse [30].	Mouse
2014	Fulka H. and Langerova	The maternal nucleolus plays a key role in centromere satellite maintenance during the oocyte to embryo transition [22].	Mouse
2014	Kyogoku et al.	NPBs start to function very soon after fertilization. Later NPBs’ enucleolation does not have a negative impact on embryonic development [26].	Mouse
2017	Morovic et al.	First successful interspecies nucleolotransfer [33].	Mouse, pig
2017	Ogushi et al.	NLB was reconstituted by single nucleolar protein Npm-2 [25].	Mouse
2020	Benc et al.	Interspecies nucleolotransfer from porcine oocyte to mouse oocyte. Interspecies quantitative differences in RPC may be compensated by multiple NLB transfer [34].	Mouse, pig

## Data Availability

No new data were created or analyzed in this study. Data sharing is not applicable to this article.
